# Molecular dynamics insights into the interfacial interactions of natural rubber and nylon 6,6 with carbon nanotubes

**DOI:** 10.1039/d6na00426a

**Published:** 2026-08-13

**Authors:** Rima Biswas, Surya S. Urs

**Affiliations:** a Process Simulation Research Group, School of Chemical Engineering, Vellore Institute of Technology Vellore Tamil Nadu India 632014 rima.biswas@vit.ac.in

## Abstract

The microscopic structure, interfacial energetics, and localized transport kinetics of natural rubber (NR) and nylon 6,6 (NY) matrices close to bare, hydroxylated (–OH), and carboxylated (–COOH) carbon nanotubes (CNTs) were examined using molecular dynamics simulations (MD). Surface functionalization significantly modifies interfacial polymer physics, as shown by radial and spatial distribution functions, which act through completely different mechanisms for polar thermoplastics and non-polar elastomers. The hierarchy of diffusivity for non-polar NR is *D*_COOH-CNT_ > *D*_OH-CNT_ > *D*_bare-CNT_. On bare CNTs, hydrophobic wetting encourages dense packing and severe mobility suppression, while polar modifications increase the local free volume by introducing steric hindrance and thermodynamic de-wetting. On the other hand, transport kinetics are completely inverted in polar NY (*D*_bare-CNT_ > *D*_OH-CNT_ > *D*_COOH-CNT_). Strong, multi-point interfacial hydrogen bonds and an intense nonbonded binding energy of −262.433 kcal mol^−1^ are established by carboxyl groups, which energetically trap the polyamide segments and significantly limit molecular hopping. Additionally, surface functionalization causes both matrices to undergo a conformational change that results in a strictly parallel alignment along the nanotube axis. These insights provide crucial design rules for tailoring interfacial shear strength and load transfer in high-performance nanocomposites.

## Abstract 2

MD simulations examined the structure, interfacial energetics, and transport kinetics of natural rubber (NR) and nylon 6,6 (NY) matrices near bare, hydroxylated, and carboxylated carbon nanotubes (CNTs).

## Graphical abstract

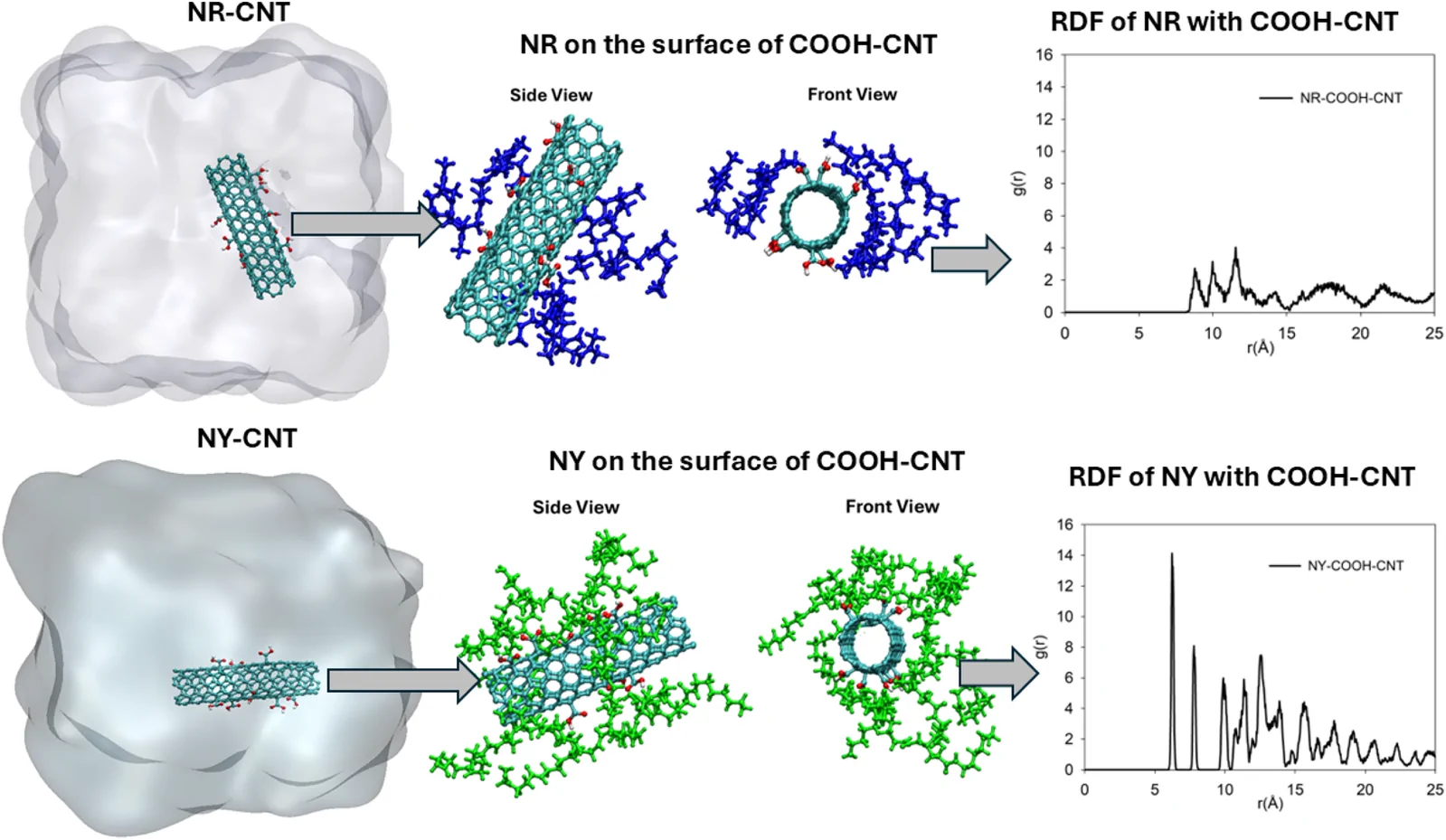

## 1. Introduction

Polymer/nanoparticle interfaces are crucial in hybrid materials because nanoparticles can modify a polymer's structural and mechanical properties without altering the matrix's chemical properties.^1,2^ CNTs are considered ideal reinforcing agents for creating high-performance, multifunctional polymer nanocomposites. This is due to their nanoscale diameter, high aspect ratio, and incredibly low density, combined with exceptional physical traits like outstanding mechanical strength and superior thermal and electrical conductivity. Experiments have thoroughly examined the characteristics of polymer/CNT composites.^3–6^ The main challenges are effectively dispersing CNTs in the polymer matrix and achieving strong interfacial interaction between the two materials.^7,8^ In order to increase the dispersion and adherence of carbon nanotubes in the polymer matrix, it is vital to pay attention to the appropriate chemical or physical treatments that may result in diverse surface morphologies. Refluxing CNTs with strong nitric acid has been shown to produce acidic sites on CNTs, including hydroxyl, carboxylic, and carbonyl groups. The combination of CNTs with the polymeric matrix will be significantly improved by these reactive groups on CNTs, increasing the mechanical strength of the nanocomposites.^9^ Sidewall functionalization also improves CNT dispersion in a polymeric matrix.^10^ To make CNTs/polymer composites, several methods have been developed, including solution-casting, melt-mixing, and *in situ* polymerization of monomers with CNTs present. The properties of polymer/CNTs composites are mostly determined by the polymers and processing techniques used to make the composites, according to Thostenson *et al.*^11^ The effects of oxatyl-modified multi-wall carbon nanotubes (MWCNTs) on the mechanical properties of epoxy composites were investigated by Guadagno *et al.*^12^ Their results indicate that the addition of unfunctionalized MWCNTs can increase the storage modulus, but functionalized nanotubes demonstrated a constant value or even a minor drop. Research on the impacts of modified CNTs is essential for the novel applications of polymer/CNT composites. Carbon-containing nylon 6 nanocomposites have been demonstrated to have remarkable properties.^13^ Gao *et al.*^14^ developed SWNT–nylon 6 composites, which possessed exceptional properties. According to their findings, a stronger SWNT–nylon interfacial connection can be formed by SWNTs with a larger concentration of carboxylic acid (–COOH) groups, which enhances the mechanical capabilities. Although NY exhibits excellent mechanical strength, thermal stability, and strong intermolecular interactions, natural rubber possesses outstanding elasticity, flexibility, and damping characteristics.

*cis*-1,4-Polyisoprene (*cis*-PI) is the primary high molecular weight polymer found in natural rubber. It is well known that NR's practical applications are limited by its poor tensile strength, low elasticity, and low abrasion resistance.^15^ However, high-performance plastics, green energy tires, and structural components of sophisticated machinery like electrical insulators and flexible sensors all make extensive use of modern rubber-based materials.^16^ Rubber technology is always being developed to create green technology with low cost and simple laboratory methods, in addition to improving physical and mechanical properties.^17^ Vulcanization is a process that improves the mechanical qualities of pure NR by heating it with sulfur. However, vulcanized NR is difficult to recycle due to sulfur cross-linking. Adding fillers like carbon black (CB) to NR-based composites can improve durability and elastic modulus.^18^ CB–NR composites exhibit significantly higher strength than vulcanized NR.^19^ Filling the NR matrix with graphene, carbon nanotubes, or fullerene (C_60_) can result in reinforced NR composites. The nanocomposites were prepared utilizing a solvent casting process, with toluene as the solvent. The study found that increasing the ratio of CNTs in nanocomposites made the rubber stronger and tougher, but also more brittle. The study found that a nanocomposite with 1 wt% CNTs had the maximum strain value. This percentage of CNTs resulted in a composite that was more ductile and elastic than others.^20^ Functionalized CNTs had a less pronounced effect. Functionalization promoted CNT dispersion in the NR matrix, which increased electrical resistance.^21^

Molecular dynamics (MD) simulations have emerged as an efficient method for studying polymer–CNT interfaces because they provide atomistic information that is difficult to get experimentally.^22–24^ Previous MD research on CNT/rubber systems has mostly focused on reinforcing mechanisms, friction behaviour, and the effects of CNT loading or geometry. For example, Li *et al.*^25^ studied the tribological behavior of CNT-reinforced nitrile butadiene rubber and found that CNT integration greatly improved shear modulus and frictional performance. Qian *et al.*^26^ investigated the effects of CNT length, content, and acrylonitrile concentration on the frictional characteristics of nitrile rubber composites. However, these investigations focused mostly on nitrile rubber and did not comprehensively study how different oxygen-containing surface functional groups control the interfacial structure and dynamics of non-polar natural rubber at the molecular level. In contrast, prior MD studies on CNT/polyamide systems have revealed that surface functionalization significantly improves interfacial adhesion. Yang *et al.*^27^ observed that the increased interfacial binding energy in functionalized CNT/nylon 6 composites is primarily due to electrostatic interactions rather than van der Waals forces. Zheng *et al.*^28^ and Frankland *et al.*^29^ also showed that chemical functionalization of CNTs can drastically alter the interfacial properties and load-transfer capability of polymer nanocomposites.

Nonetheless, this research focused mostly on interfacial binding energies and mechanical reinforcement, whereas the microscopic link between surface chemistry, polymer structure, hydrogen bond generation, and interfacial transport dynamics is still insufficiently understood. Previous molecular simulation studies have generally examined non-polar elastomers (such as NR) and polar thermoplastics (such as NY) in isolation, failing to consider how identical surface chemistries affect dramatically different polymer topologies. Furthermore, the precise atomic mechanisms dictating how oxygen-containing functional groups structurally regulate the local free volume, directional alignment, and mass transport kinetics at these various interfaces are not well defined.

To address this crucial fundamental scientific question, this study uses MD simulations to thoroughly investigate the interfacial regulation of both NR and NY matrices, which is directly mediated by the surface functionalization of single-walled carbon nanotubes (SWCNTs). By directly comparing bare, hydroxylated (–OH), and carboxylated (–COOH) CNT designs within the same computational framework, we can map the opposing thermodynamic wetting and hydrogen-bonding landscapes that govern matrix-specific segmental mobility and conformation. These fundamental microscopic insights provide clear design guidelines for modifying interfacial shear strength and load transmission in high-performance hybrid nanocomposites.

## 2. Computational details

**Fig. 1:**
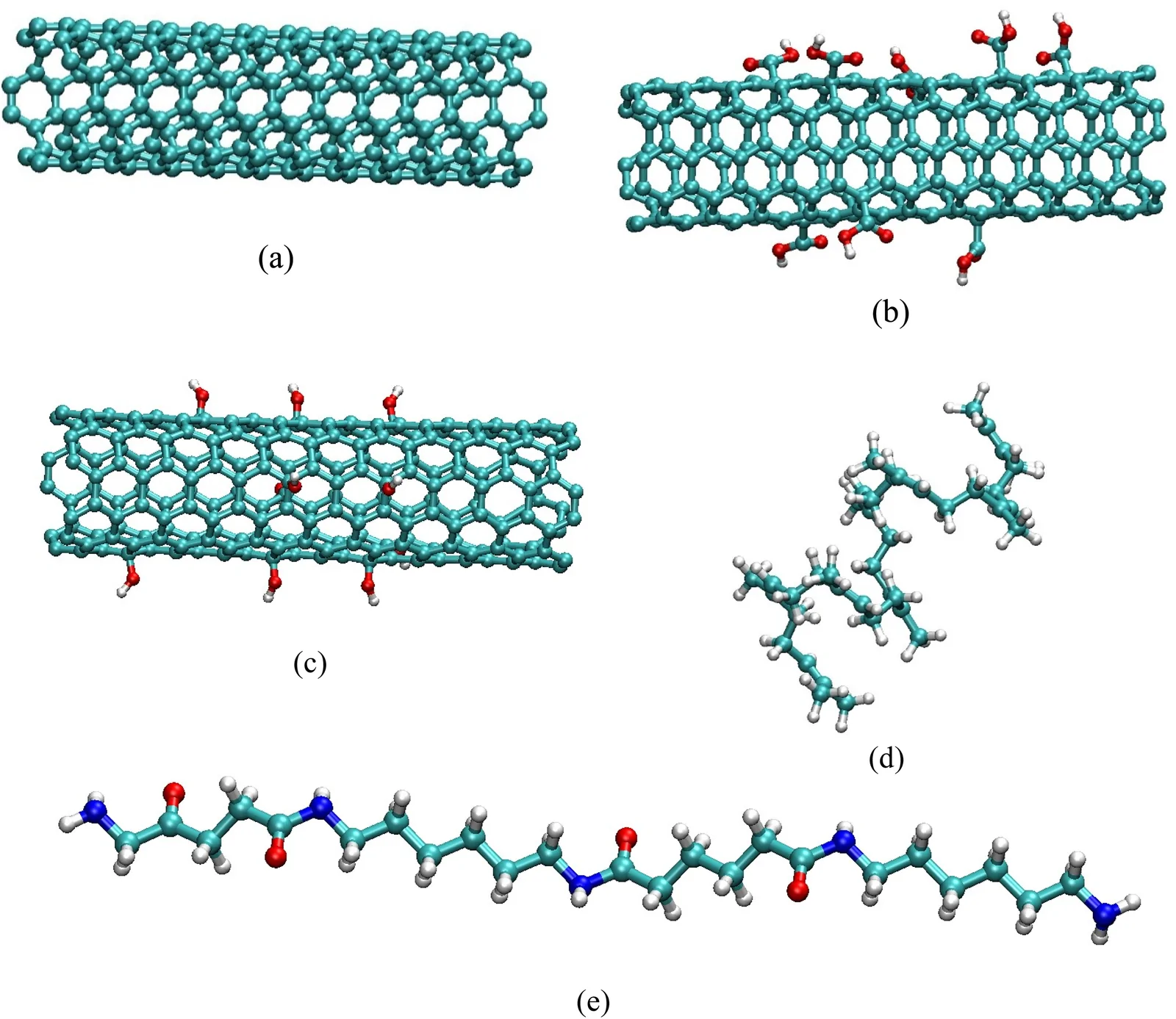
Optimization structures of (a) bare CNT, (b) COOH-CNT, (c) OH-CNT, (d) NR, and (e) NY.

The NAMD version 2.13 (ref. 30) was used for all MD simulations. The force field parameters used in this investigation were Optimized Potentials for Liquid Simulations (OPLS).^31^ The LigParGen server^32^ provided the force-field parameters of NY and natural rubber, while the bare and functionalized SWCNTs were from Anitha *et al.*^33^ We employ nylon 6,6 and natural rubber as the matrix for nanocomposite systems. In this study, single-walled carbon nanotubes (CNTs) are employed as the embedded fiber because of their superior mechanical and physical characteristics. The structural models of both pristine and functionalized single-walled carbon nanotubes (SWCNTs) were constructed using the GaussView 5.0 visualization and editing software. Subsequently, their geometry optimizations were performed with the Gaussian 09 software package, employing the B3LYP density functional theory (DFT) method alongside the Stuttgart/Dresden (SDD) basis set; the optimized configurations are illustrated in Fig. 1a–c. As shown in the optimized structures in Fig. 1b and c, a total of eight functional groups (–COOH or –OH) were uniformly connected to the sidewall of the single-walled carbon nanotube (SWCNT). With eight functional groups, the surface grafting density is approximately 1.22 groups per nm^2^. This value closely corresponds to experimental functionalization degrees in which surface defect optimization is balanced against graphitic structure retention.^34^

**Fig. 2:**
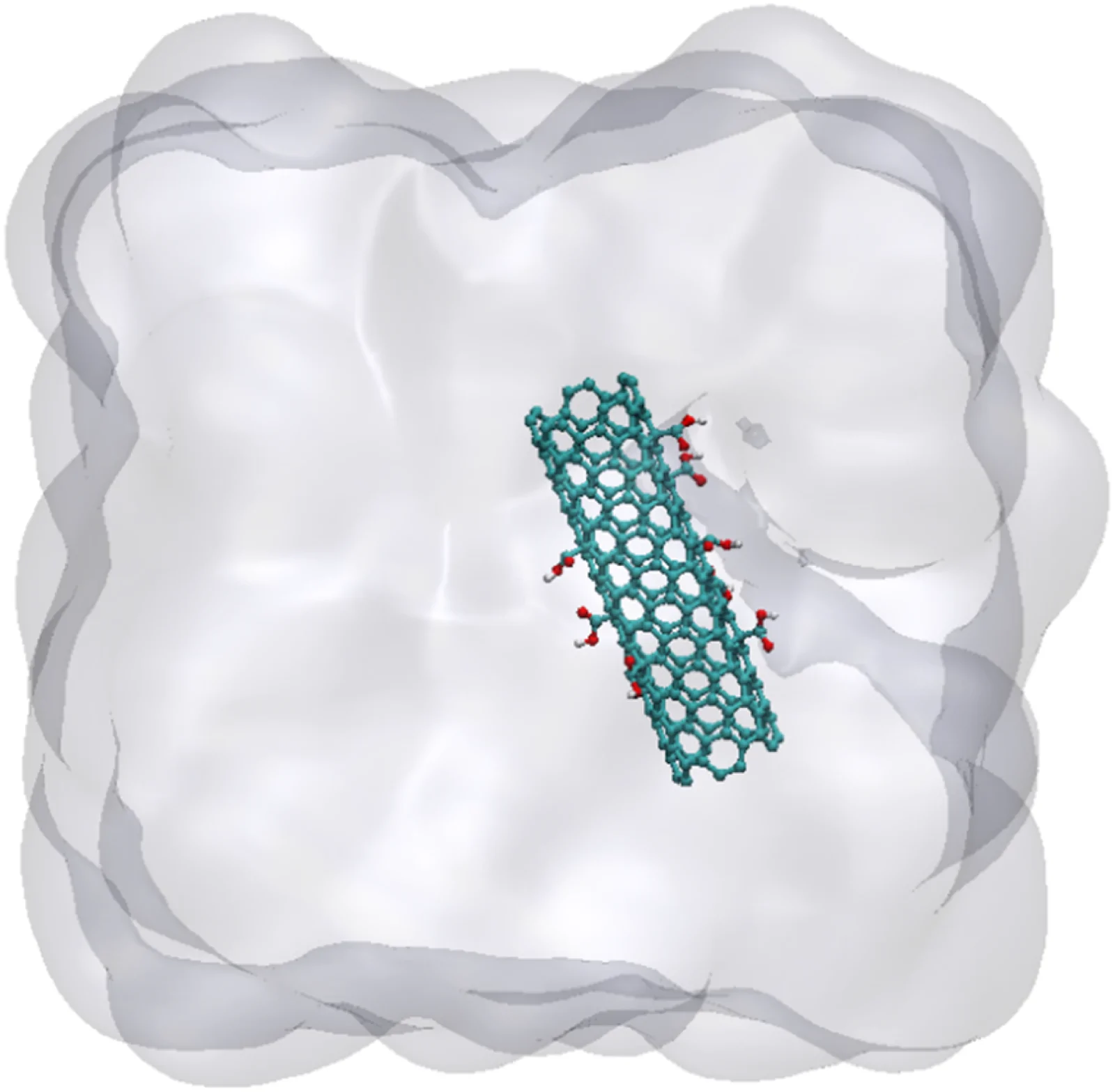
The initial snapshot of the NR–COOH functionalized CNT system.

90 short chains with 4 repeating units each make up the NY matrix (Fig. 1d), and the NR matrix is made up of 90 short chains, each of which has 8 repeating units (Fig. 1e). Short oligomeric chains were chosen largely for their computational efficiency while capturing actual unentangled local interfacial physics.^35,36^ MD simulations of the model for short-chain NY and NR in melts have demonstrated good agreement with experimental results for density.^37,38^ The study focuses on local structural ordering (RDF/SDF), binding energy profiles, and short-range transport kinetics within the immediate coordination shell (*r* < 10 Å) of the nanotube surface, so long-chain topological entanglements are not required to map the fundamental surface-energy interactions. 90 chains provided sufficient multi-chain bulk surroundings to satisfy periodic boundary conditions without significant finite-size image interaction artifacts. Each model system consists of one armchair (6, 6) SWCNTs with a length of 25.8 Å and a diameter of 8.1 Å. Our simulations make use of the periodic boundary condition. The initial cell size for the NR and NY composite systems is 55.04 × 55.04 × 55.04 Å^3^ (Fig. 2). Carbon nanotubes functionalized with –COOH have significant interfacial interactions with many polymer matrices, and the presence of –COOH functional groups on CNTs can enhance the dispersion into the matrix.^39^ Pristine or modified carbon nanotubes are used to strengthen the samples in this work. The current work examines two distinct functional groups, including two distinct oxygen-containing functional groups (–OH and –COOH). During the MD simulations, single-walled carbon nanotubes were modeled as fully flexible structures. The initial geometries of the pristine and functionalized CNTs were obtained from DFT optimization, while all bonded and non-bonded interactions during the simulations were described using the OPLS force field. There were no positional restraints were applied to the CNT atoms, allowing the nanotubes to undergo local structural relaxation in response to interactions with the surrounding polymer matrices. To get the composite systems to equilibrium, the molecular dynamics simulation with NPT ensemble is first performed for 300 ps at 1 bar and 500 K. After that, the system is gradually cooled to 250 K at a rate of 50 K/200 ps. To make sure the system reaches equilibrium at the selected temperatures, another 200 ps simulation is performed. Following the equilibration stage, a 100 ns production run was performed at 300 K. The last 10 ns of this production trajectory were used for structural and dynamical analysis to ensure that all parameters were sampled from a completely stable, fully relaxed state, with trajectory snapshots recorded every 2 ps. A 9.5 Å atom-based cut-off was used to calculate the non-bonded interactions, and the Ewald summation approach (also known as the particle mesh Ewald approximation) was used to calculate the long-range electrostatic interactions. A Langevin thermostat controlled the temperature, and a Langevin piston was used to keep an eye on the pressure. The bonds associated with hydrogen were constrained using the SHAKE method. The equations of motion were integrated using the Verlet technique with a time step of 1 fs. The equilibrated molecular dynamics trajectories were then examined to determine the structural, energetic, and dynamic properties of the polymer–CNT interfaces. Radial distribution functions (RDFs), spatial distribution functions (SDFs), non-bonded interaction energies, hydrogen-bond analyses, and mean square displacement (MSD) calculations were performed to determine the effect of CNT surface functionalization on the interfacial organization and mobility of NR and NY. These studies provide molecular-level insights into how pristine and functionalized CNT surfaces influence polymer adsorption, interfacial binding, and transport behavior. VMD software was used to capture typical trajectory snapshots.^40^ To validate the molecular model and the applicability of the OPLS-AA force field, the equilibrium densities of the simulated polymer matrices were compared with available experimental values. The calculated densities of NR and NY were 0.91 and 1.14 g cm^−3^, respectively, which agree closely with the corresponding experimental densities^37,38^ of 0.92 and 1.13 g cm^−3^. This excellent agreement suggests that the selected force field accurately represents the structural properties of both polymer matrices.

Hydrogen bond analysis was performed using the hydrogen bonds plugin in VMD. A hydrogen bond was defined using the donor–acceptor distance cutoff of 3.5 Å and a donor–hydrogen–acceptor (D–H⋯A) angle of ≥150°. These geometric criteria are commonly used in molecular dynamics studies and reliably identify intermolecular hydrogen-bonding interactions between the polymer chains and the functionalized CNT surfaces.

The Einstein equation is used to calculate the self-diffusivity of the polymers close to the CNT surface, shown in eqn (1).^41^

1 6*Dt* = 〈|*r*_*i*_(*t*) − *r*_*i*_(0)|^2^〉

Here, *r*_*i*_(*t*) and *r*_*i*_(0) are the positions of the *i*th atom at time instant *t* and at time instant 0, respectively. The self-diffusion coefficient was then calculated by the mean square displacement (MSD) plot.

## 3. Results and discussions

The structural and dynamic properties of NR and NY near the bare and functionalized CNTs are systematically investigated. We first looked at simulating the bare CNT system. Subsequently, we examined the simulation of two distinct hydrophilic group functionalized CNT systems to understand the interaction between CNT and polymers.

### 3.1. Structural properties of the simulated systems

**Fig. 3:**
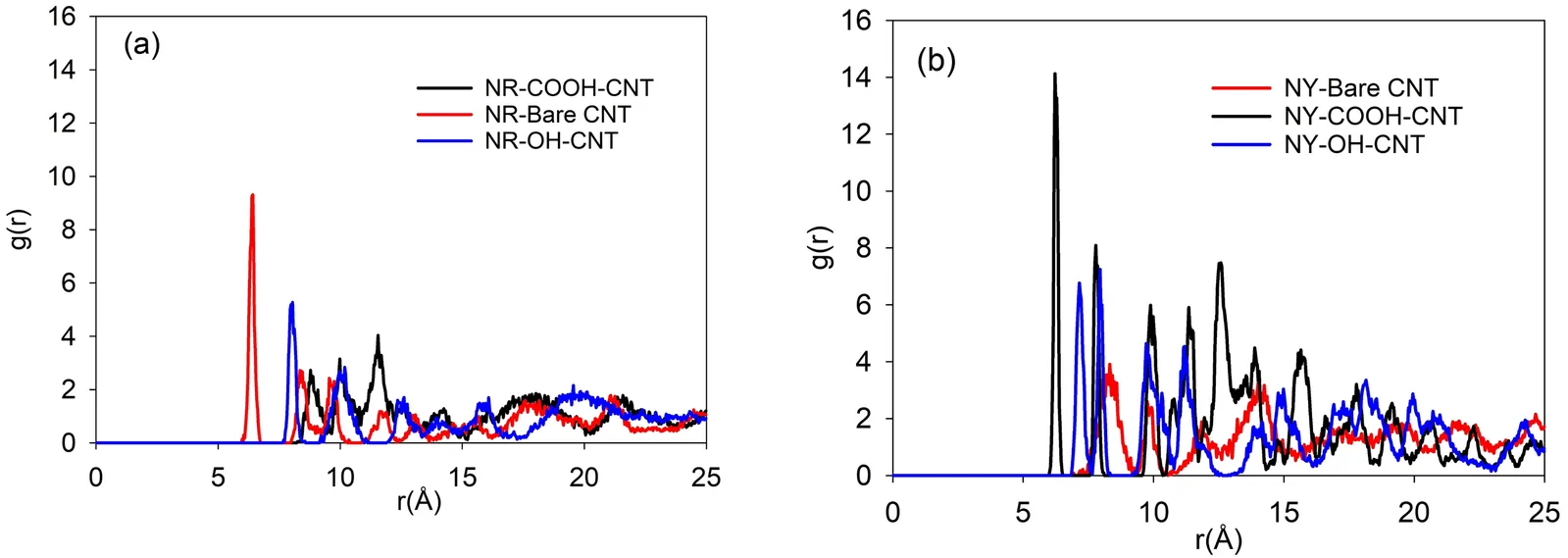
Center of mass radial distribution functions of (a) NR with bare CNT, COOH-CNT, and OH-CNT systems and (b) NY with bare CNT, COOH-CNT, and OH-CNT systems.

Understanding the interaction between polymers and CNTs requires investigating the polymer structure near the CNT surface at the microscopic level. To explore the relative positioning of NR and NY within the polymer–CNT system, radial distribution function (RDF) and spatial distribution function (SDF) analyses obtained from MD simulations were performed. To evaluate the effect of different CNT types, including bare CNT and functionalized CNTs (–COOH and –OH), the center-of-mass (COM) RDFs between NR/NY and CNT were calculated. Fig. 3 presents the RDF profiles of NR and NY with different CNT systems at 300 K. In each system, the presence of pronounced RDF peaks indicates strong interfacial interactions and electrostatic attraction between the polymers and CNTs. In the case of NR (Fig. 3a), the peak position (*r*) was observed to shift toward higher distances for functionalized CNTs (–COOH and –OH) compared to bare CNTs. Furthermore, the functionalized CNT systems exhibited significantly lower and broader first RDF peaks than the bare CNT system. This behavior indicates that the rubber chains are unable to approach the functionalized CNT surface as closely as they can with the pristine CNT surface. The broadening and flattening of the RDF peaks provide clear spatial evidence of the exclusion of polyisoprene chains from the immediate vicinity of the nanotube surface. For bare CNTs, the polyisoprene carbon atoms can approach the nanotube wall up to the van der Waals contact distance (5.98 Å). Owing to the smooth and nonpolar graphitic surface, a large fraction of polymer segments can densely pack within the first coordination shell, resulting in a sharp and intense RDF peak. In contrast, the covalently attached –COOH and –OH functional groups occupy the space adjacent to the CNT surface, generating steric hindrance that restricts the close approach of the polyisoprene backbone. Consequently, the polymer chains remain at larger distances from the nanotube surface, shifting the first RDF peak to higher distances (8.75 Å for –COOH and 7.88 Å for –OH functionalized CNTs) and reducing the peak intensity because fewer polymer segments can occupy this interfacial region. A comparison between OH-CNT and COOH-CNT further reveals that the RDF peak for OH-CNT is relatively higher and sharper than that of COOH-CNT. This difference can be attributed to the smaller steric size of the hydroxyl group. The –OH group, consisting of only one oxygen and one hydrogen atom, introduces minimal steric obstruction, allowing the polyisoprene chains to approach the graphitic surface more closely. In contrast, the bulkier –COOH group creates greater steric hindrance, thereby reducing the accessibility of the CNT surface to the NR chains.

To investigate the structural properties of NY with different CNT systems, the center-of-mass RDFs between NY and bare/functionalized CNTs were calculated. Fig. 3b shows the RDF profiles of NY with bare CNT, COOH-CNT, and OH-CNT systems at 300 K. In Fig. 3b, the NY chains had the closest proximity to COOH-CNT, with the first RDF peak at *r* = 6.215 Å, followed by OH-CNT at *r* = 7.160 Å. This behavior is principally determined by the strong hydrogen bonding affinity between NY and the functional groups on the CNT surface. The carboxyl group has more hydrogen-bonding capability and a larger electrostatic field than the hydroxyl group (–OH), allowing for stronger and more stable interfacial contacts with the NY matrix. As a result, the NY chains are more firmly adsorbed and structurally structured around the COOH-functionalized CNT surface, producing a sharper and more intense RDF peak. In the case of OH-CNT, hydrogen bonding is still present but comparatively weaker, resulting in slightly lower structural ordering. In contrast, bare CNTs lack polar functional groups and therefore interact with NY mainly through weaker van der Waals interactions. Consequently, the NY chains remain relatively mobile and continuously fluctuate around the CNT surface at varying distances, producing a broader and less intense RDF peak.

**Fig. 4:**
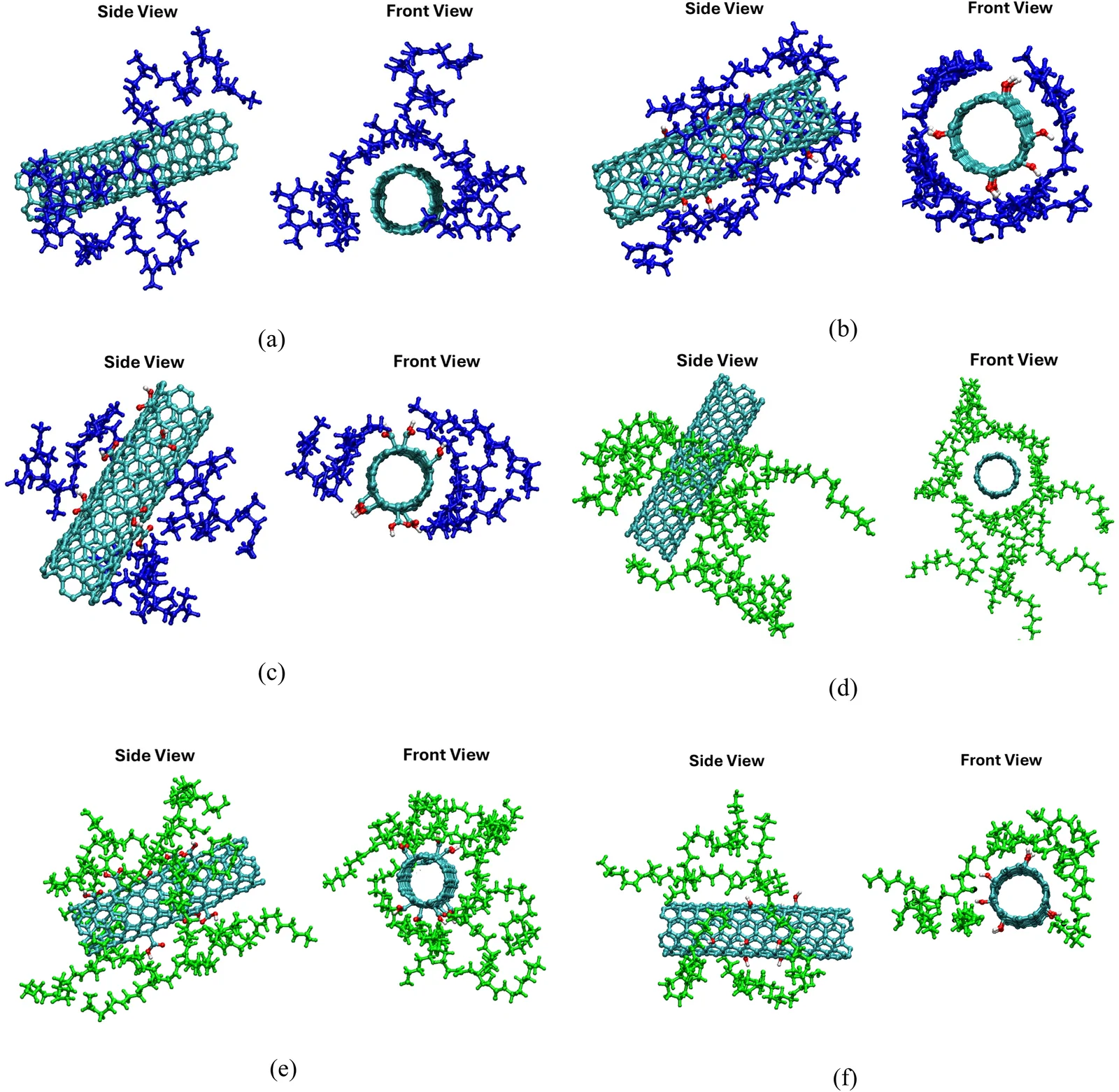
The orientations of NR (blue) near the interface of the (a) bare CNT, (b) COOH-CNT, and (c) OH-CNT, and NY (green) near the interface of the (d) bare CNT, (e) COOH-CNT, and (f) OH-CNT.

**Fig. 5:**
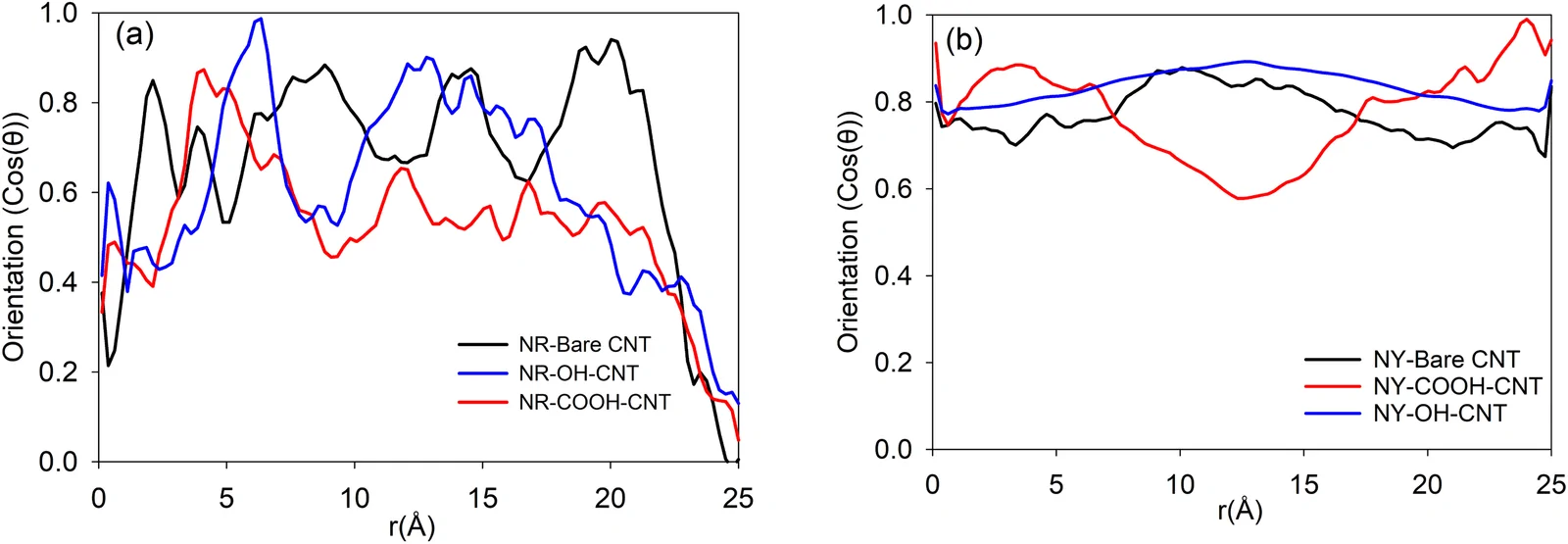
Ordering orientation of (a) NR and (b) NY around the CNT as a function of radial distance.

Fig. 4 illustrates the conformational orientations of natural rubber and nylon 6,6 near the CNT surfaces. On the pristine CNT surface, NR exhibits a combination of parallel and perpendicular alignments (Fig. 4a), which is quantitatively validated by the sharp local minimum (〈cos(*θ*)〉 ≈ 0.22) at the immediate interface (*r* < 1 Å) in Fig. 5a. While the long polymer backbones predominantly align parallel to the bare nanotube axis as evidenced by the subsequent major peak (∼0.85) at *r* = 2 Å, a few NR segments adopt a perpendicular orientation, driven by localized CH–π interactions that drive the rapid spatial oscillations in the pristine profile. In contrast, upon surface functionalization with –COOH and –OH groups, the NR chains preferentially adopt a strictly parallel orientation (Fig. 4b and c). This structural transition is clearly reflected in the orientation profiles, where functionalization eliminates the deep interfacial dip seen in the bare system, maintaining significantly higher 〈cos(*θ*)〉 values near the surface and forcing a highly ordered parallel packing peak (∼1.0) for the hydroxylated system near 6 Å. A similar orientational transition is observed for NY. On the bare CNT, a small fraction of NY chains display a mixed alignment of perpendicular and parallel orientations (Fig. 4d), as quantitatively confirmed by the local orientation dip (〈cos(*θ*)〉 ≈ 0.74) at the immediate interface (*r* < 1 Å) in Fig. 5b. However, when interacting with COOH- and OH-functionalized CNT surfaces, the NY chains uniformly undergo a structural rearrangement to adopt a strict parallel configuration (Fig. 4e and f). This transition is mathematically supported by the high interfacial baseline values in Fig. 5b, where the hydroxylated and carboylated systems exhibit a significantly stronger parallel anchoring (〈cos(*θ*)〉 ≈ 0.84 and 0.94, respectively) directly at the surface. This favorable interfacial wetting effectively suppresses the localized conformational tilting observed on the pristine nanotube. This transition from a mixed alignment to highly ordered parallel polymer wrapping shells directly follows established molecular dynamics precedents for nanotube templates.^4,22^

**Fig. 6:**
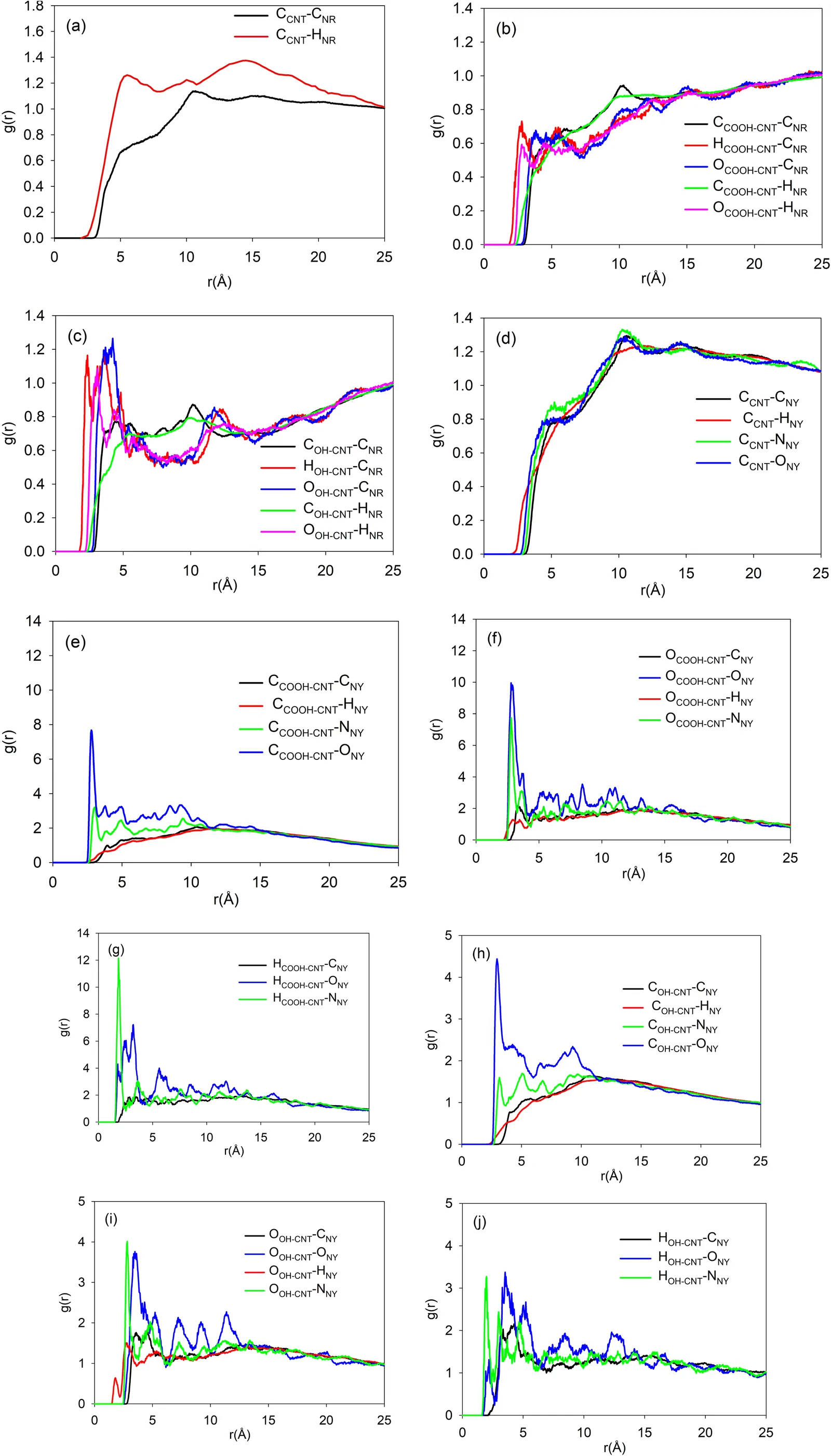
Atomic radial distribution functions for (a) C_CNT_–C_NR_ and C_CNT_–H_NR_ in bare CNT–NR system, (b) C_COOH-CNT_–C_NR_, H_COOH-CNT_–C_NR_, O_COOH-CNT_–C_NR_, C_COOH-CNT_–H_NR_, and O_COOH-CNT_–H_NR_ in COOH-CNT–NR system, (c) C_OH-CNT_–C_NR_, H_OH-CNT_–C_NR_, O_OH-CNT_–C_NR_, C_OH-CNT_–H_NR_, and O_OH-CNT_–H_NR_ in OH-CNT–NR system, (d) C_CNT_–C_NY_, C_CNT_–H_NY_, C_CNT_–N_NY_, C_CNT_–O_NY_ in bare CNT–NY system, (e) C_COOH-CNT_–C_NY_, C_COOH-CNT_–H_NY_, C_COOH-CNT_–N_NY_, and C_COOH-CNT_–O_NY_ in COOH-CNT–NY system, (f) O_COOH-CNT_–C_NY_, O_COOH-CNT_–O_NY_, O_COOH-CNT_–H_NY_, and O_COOH-CNT_–N_NY_ in COOH-CNT–NY system, (g) H_COOH-CNT_–C_NY_, H_COOH-CNT_–O_NY_, and H_COOH-CNT_–N_NY_ in COOH-CNT–NY system, (h) C_OH-CNT_–C_NY_, C_OH-CNT_–H_NY_, C_OH-CNT_–N_NY_, and C_OH-CNT_–O_NY_ in OH-CNT–NY system, (i) O_OH-CNT_–C_NY_, O_OH-CNT_–O_NY_, O_OH-CNT_–H_NY_, and O_OH-CNT_–N_NY_ in OH-CNT–NY system, and (j) H_OH-CNT_–C_NY_, H_OH-CNT_–O_NY_, and H_OH-CNT_–N_NY_ in OH-CNT–NY system.

To understand the arrangement of polymer molecules around the CNTs, the atomic radial distribution functions (RDFs) were calculated for the polymer–CNT interactions. Fig. 6 displays the atomic RDFs for the interactions of NR and NY with the CNTs. As shown in Fig. 6a, a closer association was observed between the hydrogen atoms of NR (H_NR_) and the carbon atoms of the bare CNT (C_CNT_), exhibiting an RDF peak at 5.20 Å, compared to the broad peak corresponding to the NR carbon atoms (C_NR_). This result suggests that while the hydrogen atom directly interacts with the CNT carbon to satisfy the geometry of the CH–π interaction, the carbon atom positioned immediately behind it provides the heavy thermodynamic stability that keeps the rubber chain bound to the bare nanotube surface. In the case of the COOH-functionalized CNT (COOH-CNT) system, the RDF between C_NR_ and the hydrogen atom of the COOH-CNT (H_COOH-CNT_) shows an initial peak at 2.60 Å. This close association of C_NR_ with H_COOH-CNT_ is illustrated in Fig. 6b, which is followed by the interactions of O_COOH-CNT_ with the hydrogen (H_NR_) and carbon (C_NR_) atoms of NR, whose peaks are located at 2.71 Å and 3.82 Å, respectively. Similarly, Fig. 6c shows the atomic RDFs for the primary interactions of the hydroxyl-functionalized CNT (OH-CNT) system. A close association was found between the hydrogen atom of OH-CNT (H_OH-CNT_) and C_NR_, with an RDF peak at 2.31 Å, followed by the interactions of O_OH-CNT_–H_NR_ and O_OH-CNT_–C_NR_ at 3.09 Å and 3.57 Å, respectively.

On the other hand, the RDFs between the carbon atoms of the bare CNT (C_CNT_) and the oxygen (O_NY_) and nitrogen (N_NY_) atoms of NY show a closer association, with RDF peaks at 4.68 Å and 5.10 Å, respectively. As shown in Fig. 6d, these are followed by the interactions of C_CNT_ with the hydrogen (H_NY_) and carbon (C_NY_) atoms of NY. In the COOH-CNT system, a closer association was found between the carbon atoms of the COOH-CNT (C_COOH-CNT_) and the (O_NY_) and (N_NY_) atoms of NY, which exhibit RDF peaks at 2.74 Å and 3.02 Å, respectively, outperforming the other interaction peaks (Fig. 6e). Notably, the RDF between the hydrogen atom of the COOH group (H_COOH-CNT_) and the nitrogen atom of NY (N_NY_) displays the sharpest initial peak at 1.87 Å (Fig. 6f). Furthermore, the RDF between the oxygen atom of the COOH group (O_COOH-CNT_) and the oxygen or nitrogen atoms of NY shows a sharp peak at 2.81 Å, indicating a strong interaction within the COOH-CNT/NY system (Fig. 6g). Finally, from Fig. 6h–j, it can be concluded that the carbon (C_OH-CNT_), oxygen (O_OH-CNT_), and hydrogen (H_OH-CNT_) atoms of the OH-CNT are situated closer to the (O_NY_) and (N_NY_) atoms of NY than to its other constituents.

**Fig. 7:**
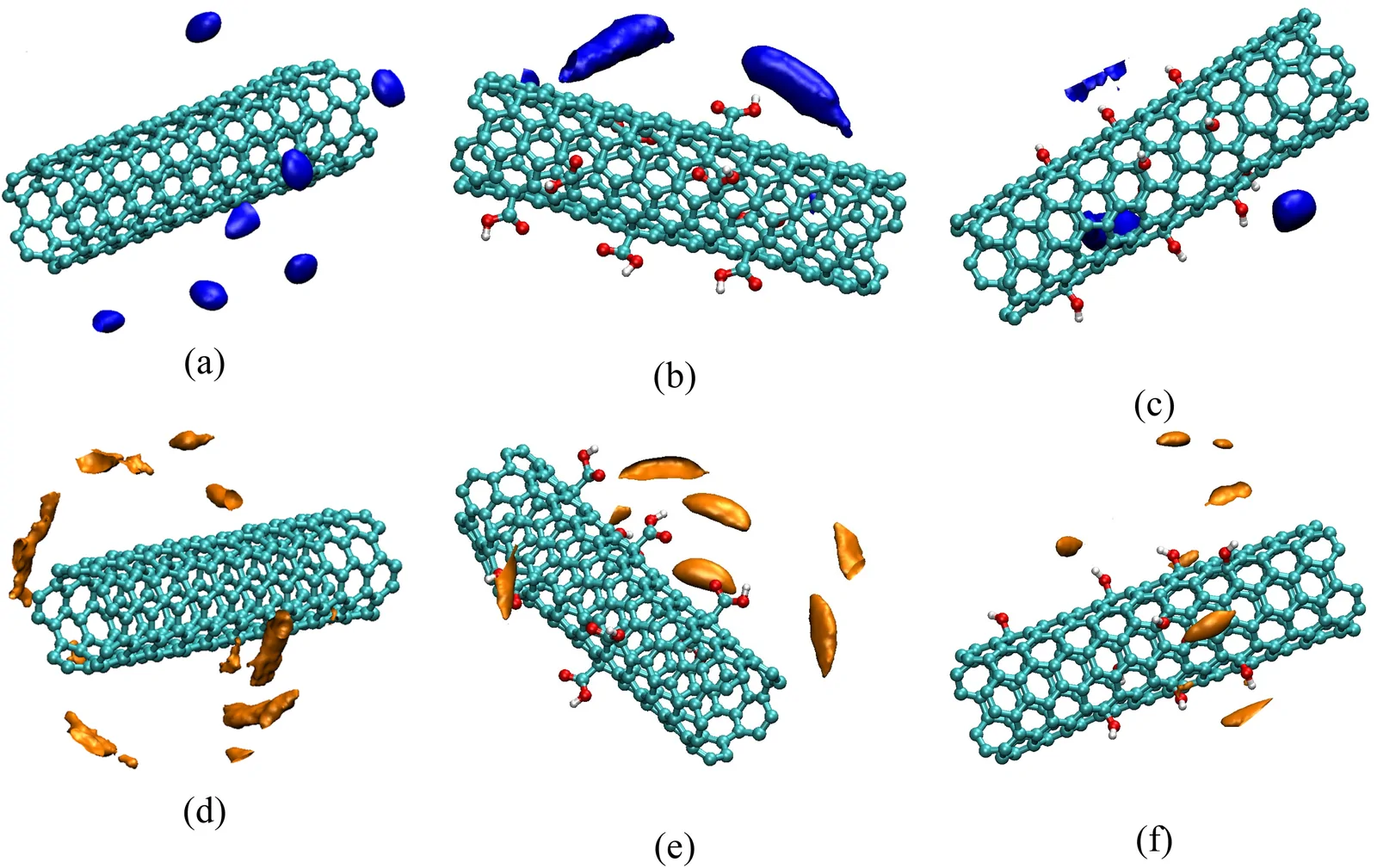
Spatial distribution functions of NR around (a) bare CNT, (b) COOH-CNT, (c) OH-CNT, and NY around (d) bare CNT, (e) COOH-CNT, and (f) OH-CNT.

As shown in Fig. 7, the spatial distribution function (SDF) was computed to further investigate the arrangement of polymer molecules around the CNT surface. The significant local organization of polymer molecules around the CNT highlights the importance of the interaction type between the polymer and CNT. The SDFs were calculated using the TRAVIS software.^42^ In the case of NR with bare CNT, the NR isosurface appears near the CNT surface, indicating that NR interacts with the bare CNT within the first solvation shell (Fig. 7a). The isosurfaces corresponding to NR and NY are represented by blue and orange colors, respectively, in Fig. 7a–f. A similar arrangement of NR was observed around both COOH-functionalized CNT and OH-functionalized CNT, where the average density distribution of NR was found to be higher near the carboxyl group (Fig. 7b) and hydroxyl group (Fig. 7c) on the functionalized CNT surfaces. To further examine the average density distribution of NY around the CNTs, the SDFs of NY surrounding bare CNT, COOH-CNT, and OH-CNT were also determined, as shown in Fig. 7d–f. It was observed that the average density distribution of NY around COOH-CNT (Fig. 7e) was higher than that around OH-CNT (Fig. 7f) and bare CNT (Fig. 7d). This observation is further supported by the higher RDF peak heights obtained for the NY/COOH-CNT system (Fig. 3b). Moreover, calculating the interaction energy can provide additional insight into the strength of the interfacial interactions between the polymer and CNT systems.

### 3.2. Interaction energy

Table 1 presents the nonbonded interaction energies-specifically the electrostatic and van der Waals (vdW) components-between the respective polymers and the CNTs. These energies were computed using the NAMD Energy plugin within the VMD software suite. To ensure optimal mesh density and an accurate assessment of long-range electrostatic interactions, calculations were performed using periodic boundary conditions coupled with the Particle Mesh Ewald (PME) method.^43^ The resulting interfacial energies provide important information about how the CNTs interact with the natural rubber and nylon 6,6 matrices. Interestingly, compared to the OH-functionalized (−68.742 kcal mol^−1^) and COOH-functionalized (−50.135 kcal mol^−1^) versions, the total interaction energy between NR and the bare CNT (−98.287 kcal mol^−1^) was noticeably stronger. Deconstruction of these values reveals that NR and a bare CNT have no net electrostatic interaction in the classical sense, yielding an *E*_elec_ value of 0.000 kcal mol^−1^. The interaction energy between the bare CNT and NR is solely controlled by vdW forces (*E*_vdW_ = −98.287 kcal mol^−1^) since both components are totally non-polar and lack localized charges, which prevents them from producing persistent coulombic forces. Pristine, bare CNTs have a highly delocalized electron network throughout their whole surface, making the cylinder extremely polarizable. London dispersion forces are optimized over a wide surface area and close atomic interaction is maximized when the very flexible hydrocarbon chains of the NR matrix adhere to this smooth topography. On the other hand, the CNT surface develops highly polar, hydrophilic zones when COOH and OH functional groups are added. The local polymer–nanotube interface is thermodynamically and energetically unfavorable because the non-polar rubber chains have no equivalent dipoles to satisfy these polar patches; hence, the electrostatic contribution is completely muted (0.000 kcal mol^−1^). This mismatch causes the rubber backbones to structurally disfavor and avoid the functionalized patches, introducing a severe steric penalty that forces a drop in the van der Waals attraction forces to −68.742 kcal mol^−1^ for the OH-CNT and a minimum of −50.135 kcal mol^−1^ for the COOH-CNT system.

**Table 1:** The nonbonded interaction energies of NR and NY with bare CNT, COOH-CNT, and OH-CNT

Systems	Electrostatic (*E*_elec_) (kcal mol^−1^)	van der Waals (*E*_vdW_) (kcal mol^−1^)	Total (kcal mol^−1^)
NR–bare CNT	0.000	−98.287	−98.287
NR–OH-CNT	0.000	−68.742	−68.742
NR–COOH-CNT	0.000	−50.135	−50.135
NY–bare CNT	0.000	−139.730	−139.730
NY–OH-CNT	−53.175	−122.729	−175.904
NY–COOH-CNT	−68.463	−193.970	−262.433

The overall interaction energy for the NY matrix showed a different pattern: the COOH-functionalized CNT showed the greatest binding (−262.433 kcal mol^−1^), which was followed by the OH-functionalized version (−175.904 kcal mol^−1^) and the bare CNT (−139.730 kcal mol^−1^). Isolating the components indicates that the intense structural affinity of the COOH-functionalized surface is heavily influenced by an exceptional electrostatic contribution (*E*_elec_ = −68.463 kcal mol^−1^) associated with an expanded van der Waals energy of −193.970 kcal mol^−1^.

The dual structural features of the carboxylic acid group, which have a hydroxyl donor (–OH) and a carbonyl acceptor (–C=O), account for the remarkable affinity of the COOH-functionalized surface. This allows the –COOH group to form highly stable, cooperative hydrogen-bonded pairs with the repeating amide groups of the NY backbone; specifically, the hydroxyl proton of the acid coordinates with the amide carbonyl, while the amide –NH donor simultaneously couples with the acid carbonyl. Under dense local configurations or interfacial stress, this interaction can exhibit partial ionic character due to localized proton sharing with the amide nitrogens. This highly synergistic, dual-site alignment rigidly anchors the polymer chains to the functionalized sites, driving the total interfacial energy down to its lowest value of −262.433 kcal mol^−1^. This strong binding behavior is consistent with prior energy component analyses of polyamide matrices, indicating that polar surface treatments shift the matrix-filler interaction paradigm from a dispersion-dominated to an electrostatic-dominated regime.^27,28^ Conversely, the OH-functionalized CNT lacks the highly polar carbonyl companion found in the –COOH group. While the single hydroxyl group is fully capable of establishing robust hydrogen bonds with the NY chains-rendering its total interaction energy significantly more favorable than that of the pristine tube-it lacks the multi-point coordination density of the carboxylic acid. Finally, the interface with the bare CNT yields the weakest total interaction energy (−139.730 kcal mol^−1^). Because the pristine carbon lattice offers no polar or complementary hydrogen-bonding sites to satisfy the amide groups (*E*_elec_ = 0.000 kcal mol^−1^), the interface is governed strictly by weak van der Waals dispersion forces acting on the aliphatic methylene (–CH_2_–) segments of the polymer. Furthermore, the rigid, planar conformation of the amide linkages induces a minor steric penalty, slightly hindering the NY backbone from wrapping as flatly or densely against the cylindrical carbon lattice as a purely flexible hydrocarbon chain.

**Fig. 8:**
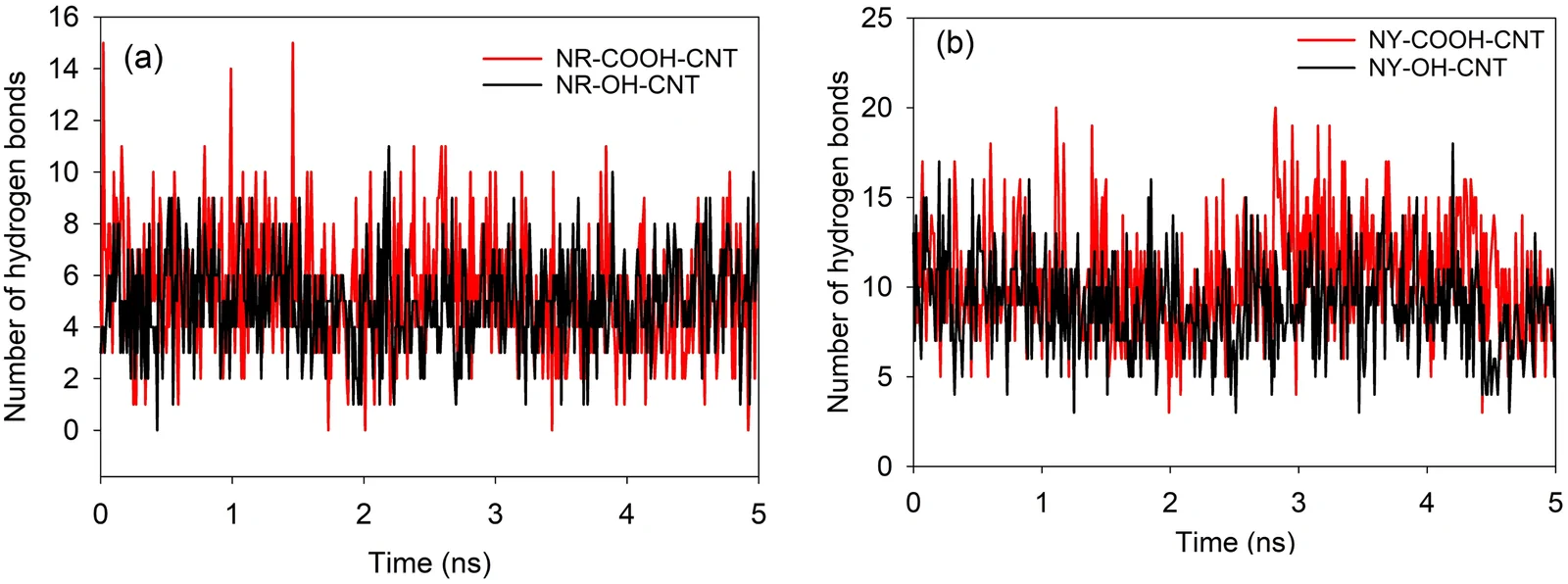
Average number of hydrogen bonds observed in (a) NR with COOH-CNT, and OH-CNT systems, (b) NY systems with COOH-CNT, and OH-CNT systems.

Fig. 8 illustrates the temporal evolution of hydrogen bonds across the simulated systems. Interfacial hydrogen bonding occurs in the following order within the natural rubber (NR) matrix: COOH-CNT > OH-CNT > bare CNT. Filler self-association is responsible for this behavior; the –OH groups form typical linear hydrogen bonds, which are intrinsically weaker than the acid dimers, while the –COOH groups form remarkably stable, cyclic hydrogen-bonded dimers with one another (Fig. 8a). The addition of functionalized CNTs fails to create advantageous interactions with the matrix because NR lacks polar functional groups. As a result, the average hydrogen bond count is much lower than in NY systems. On the other hand, NY is a polyamide rich in highly polar amide linkages (–CONH) with hydrogen bond donors (–NH) and acceptors (C=O) along its repeating backbone. This molecular architecture enables full interfacial hydrogen bonding, although the hierarchy remains COOH-CNT > OH-CNT > bare CNT (Fig. 8b). The –COOH groups exhibit exceptionally strong interactions with the amide groups of NY: the carboxylic acid's acidic proton establishes a strong hydrogen bond with the polymer backbone's highly electronegative carbonyl oxygen (C=O), while the acid's carbonyl oxygen concurrently takes up a hydrogen bond from the NY's –NH– group. Interfacial shear strength and load transfer inside the composite are significantly increased by this dual-interaction process, which creates an incredibly robust and chemically compatible interface. While hydroxyl groups (–OH) also form genuine interfacial hydrogen bonds with NY, they are significantly weaker than those mediated by the carboxylic acids. Finally, the pristine, bare CNT retains a pure sp^2^ carbon lattice completely devoid of polar functional groups, rendering it chemically incapable of forming hydrogen bonds with either matrix.

### 3.3. Interfacial diffusivity of polymer chains near the CNT surface

**Fig. 9:**
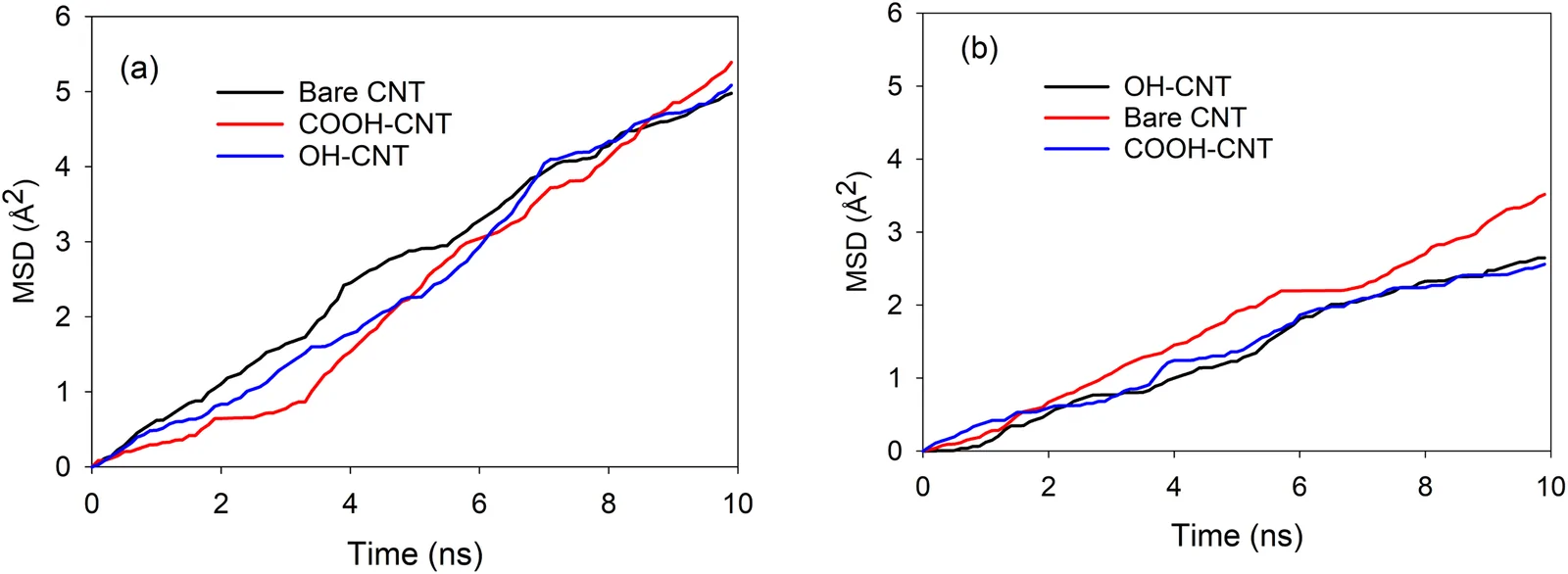
MSD of (a) NR in bare CNT, COOH-CNT, and OH-CNT systems and (b) NY in bare CNT, COOH-CNT, and OH-CNT systems.

The self-diffusion coefficient of the species in the medium, which is theoretically explained by the Einstein equation,^44^ defines the mobility (*i.e.*, rate of mass transfer) of a molecule. The self-diffusion coefficients for NR and NY near the CNT have been measured using the mean square displacement profiles, as shown in Fig. 9. The diffusion coefficient of natural rubber near the bare CNT is found to be lower than that of COOH-CNT and OH-CNT, as mentioned in Table 2. The smooth, unfunctionalized graphitic surface of a bare CNT is highly hydrophobic and non-polar. It shares a strong thermodynamic affinity with the non-polar, hydrophobic methyl and methylene groups of natural rubber. This leads to tight interfacial packing (“good wetting”), strong local van der Waals adsorption, and a severe drop in NR diffusivity. Introducing highly polar carboxyl or hydroxyl groups completely alters the surface energy. The non-polar natural rubber chains experience poor thermodynamic affinity with these polar patches. This incompatibility causes a subtle local “de-wetting” effect or loose packing at the atomic interface. Because the polymer is not tightly bound or strongly adsorbed to the functional sites, it retains higher segmental mobility and higher diffusivity. Carboxyl groups on the CNT surface can form strong, dimeric hydrogen-bonded networks with neighboring –COOH groups. When these groups lock onto each other, they effectively “shield” parts of the CNT surface and reduce the surface area available to interact with the polymer, allowing the non-polar natural rubber to glide past more freely. While natural rubber is a non-polar hydrocarbon, NY is a highly polar polyamide containing strongly electronegative carbonyl groups (C=O) and amine groups (N–H). The diffusion coefficient of NY near the CNT surface follows the opposite trend: *D*_bare-CNT_ > *D*_OH-CNT_ > *D*_COOH-CNT_. This is directly attributable to the formation of localized, high-energy interfacial hydrogen bonds between the polyamide backbone and the oxygen-bearing surface groups (–OH and –COOH). The strong electrostatic attractions act as physical anchoring sites that heavily restrict segmental hopping. The bulkier, more polar –COOH groups establish a more robust hydrogen-bonding network with the amide linkages than the –OH groups, culminating in the lowest diffusion coefficients at the COOH-CNT interface. This complete inversion of mass transport kinetics is consistent with atomistic studies demonstrating that oxygenated surface patches control the localized jumping barriers and segmental friction coefficients of surrounding chains.^23,36^

**Table 2:** Diffusion coefficients of NR and NY in bare CNT, COOH-CNT, and OH-CNT systems

Systems	Diffusion coefficient (*D* × 10^−11^ m^2^ s^−1^)
NR–bare CNT	4.624
NR–COOH-CNT	5.639
NR–OH-CNT	5.174
NY–bare CNT	2.933
NY–COOH-CNT	2.206
NY–OH-CNT	2.704

## 4. Conclusions

In this study, MD simulations were successfully deployed to systematically investigate the microscopic structure, interfacial energetics, and localized transport dynamics of NR and NY matrices in the vicinity of bare, OH-functionalized, and COOH-functionalized CNTs. The computational insights reveal that surface functionalization fundamentally alters the interfacial polymer physics, operating through entirely opposing mechanisms for non-polar elastomers *versus* polar thermoplastics. Spatial and conformational analyses demonstrate that surface functionalization forces both flexible hydrocarbon backbones and rigid amide segments to undergo an orientational transition from a hybrid parallel/perpendicular arrangement on bare CNTs into a strictly uniform, parallel configuration along the functionalized nanotube walls to either minimize unfavorable polar contact (NR) or maximize multi-point interfacial hydrogen bonding (NY). The order of interfacial self-diffusivity for NR is *D*_COOH-CNT_ > *D*_OH-CNT_ > *D*_bare-CNT_. Hydrophobic wetting and advantageous van der Waals contacts increase compact atomic packing within the initial coordination shell on the pure, non-polar graphitic surface of bare carbon nanotubes (CNTs), significantly reducing segmental mobility. On the other hand, steric barrier and thermodynamic incompatibility are introduced when polar functional groups are added. The non-polar polyisoprene chains can move beyond the nanotube surface with increased diffusivity because the bulkier –COOH groups break up regular concentric polymer packing, increasing the local free volume at the interface. The localized transport kinetics show a full inversion for the NY matrix. Strong electrostatic attraction and localized potential energy traps control this activity. The development of a very dense, stiff adsorption layer surrounding COOH-CNT, supported by a strong interfacial hydrogen bond, is clearly captured by RDFs and atomic tracking. These findings provide a fundamental framework for the rational design of polymer nanocomposites. The precise quantification of how surface chemistry controls the trade-off between interfacial binding energy and segmental mobility offers crucial guidelines for tailoring macro-mechanical properties. Specifically, while pristine CNTs are thermodynamically optimized for flexible, non-polar elastomeric matrices, carboxyl-functionalization is highly recommended for polar polyamide composites to establish ultra-rigid interphase layers capable of maximizing interfacial shear strength and load transfer.

## Author contributions

Rima Biswas: writing – review and editing, supervision, validation, investigation, formal analysis, data collection, visualization, methodology, conceptualization. Surya S. Urs: writing – original draft, methodology, investigation, formal analysis, data collection, visualization, conceptualization.

## Conflicts of interest

There are no conflicts to declare.

## Data Availability

The datasets used and/or analysed during the current study available from the corresponding author on reasonable request.
